# Periostin: a promising target of therapeutical intervention for prostate cancer

**DOI:** 10.1186/1479-5876-9-99

**Published:** 2011-06-30

**Authors:** Chuanyu Sun, Xiaojun Zhao, Ke Xu, Jian Gong, Weiwei Liu, Weihong Ding, Yuancheng Gou, Guowei Xia, Qiang Ding

**Affiliations:** 1 Department of Urology, Huashan Hospital, Fudan University, Shanghai, 200040 China; 2 The Central Laboratory, Yueyang Hospital of Intergrated Traditional Chinese and Western Medicine, Shanghai University of Traditional Chinese Medicine, Shanghai, 200437, China; 3 Department of Laboratory Medicine, Huashan Hospital, Fudan University, Shanghai, 200040 China

**Keywords:** Periostin, Prostate cancer, RNAi, Proliferation, Migration

## Abstract

**Background:**

In our recent study, Periostin was up-regulated in prostate cancer(PCa) compared with benign prostate hyperplasia (BPH) by proteomics analysis of prostate biopsies. We investigated the effect of sliencing Periostin by RNA interference (RNAi) on the proliferation and migration of PCa LNCap cell line.

**Methods:**

All the prostate biopsies from PCa, BPH and BPH with local prostatic intraepithelial neoplasm(PIN) were analyzed by iTRAQ(Isobaric tags for relative and absolute quantification) technology. Western blotting and immunohistochemical staining were used to verify Periostin expression in the tissues of PCa. Periostin expression in different PCa cell lines was determined by immunofluorescence staining, western blotting and reverse transcription PCR(RT-PCR). The LNCap cells with Periostin expression were used for transfecting shRNA-Periostin lentiviral particles. The efficancy of transfecting shRNA lentiviral particles was evaluated by immunofluorescence, western blotting and Real-time PCR. The effect of silencing Periostin expression by RNAi on proliferation of LNCap cells was determined by MTT assay and tumor xenografts. The tissue slices from theses xenografts were analyzed by hematoxylin and eosin(HE) staining. The expression of Periostin in the xenografts was deteminned by Immunohistochemical staining and western blotting. The migration of LNCap cells after silencing Periostin gene expression were analyzed in vitro.

**Results:**

Periostin as the protein of interest was shown 9.12 fold up-regulation in PCa compared with BPH. The overexpression of Periostin in the stroma of PCa was confirmed by western blotting and immunohistochemical staining. Periostin was only expressed in PCa LNCap cell line. Our results indicated that the transfection ratio was more than 90%. As was expected, both the protein level and mRNA level of Periostin in the stably expressing shRNA-Periostin LNCap cells were significantly reduced. The stably expressing shRNA-Periostin LNCap cells growed slowly in vitro and in vivo. The tissues of xenografts as PCa were verificated by HE staining. Additionally, the weak positive Periostin expressed tumor cells could be seen in the tissues of 6 xenografts from the group of down-regulated Periostin LNCap cells which had a significant decrease of the amount of Periostin compared to the other two group. Furthermore, our results demonstrated that sliencing Periostin could inhibit migration of LNCap cells in vitro.

**Conclusions:**

Our data indicates that Periostin as an up-regulated protein in PCa may be a promising target of therapeutical intervention for PCa in future.

## Background

Periostin, also named osteoblast-specific factor 2, was initially identified as a secreted extracellular matrix protein in the mouse osteoblastic MC3T3-E1cell line[1]. The sequence of Periostin contains a typical signal sequence, a cysteine-rich domain, a fourfold fasciclin 1-like (FAS-1) domain and a C-terminal domain[1,2].The FAS-1 domain, an evolutionarily ancient adhesion domain, also exists in many proteins such as big-h3, stabling I and II, MBP-70, algal-CAM and Periostin-like factor. Therefore, all these proteins including Periostin with the FAS-1 domain belong to the fasciclin family[3]. Additionally, Periostin shares high homology in human and mouse species: 89.2% amino acid identity in total and 90.1% identity in their mature forms[4]. Periostin gene is located on chromosome 3 in mouse compared with chromosome 13q in human which encodes a Periostin of 835 amino acids with a MW of 90 kDa[5].

Periostin can interact with other extracelluar matrix proteins such as fibronectin, tenascin C, collagen type I, collagen type V and heparin. And, it can induce integrin-dependent cell adhesion and motility by binding to αvβ3 or αvβ5 integrins[6]. Periostin is highly expressed in many normal tissues such as periosteum, perichondrium, periodontal ligaments, the fascia of muscles, articular surfaces of the epiphyseal cartilage and joint ligaments[7-9]. Thus, it is perceived as playing a potential role in the formation and structural maintenance of all these tissues[9]. Additionally, it has been reported that the expression of Periostin is correlated with the development of the heart and some heart diseases[10,11].

Recently, The overexpression of Periostin has been found in various human cancers including non-small-cell lung cancer, ovarian cancer, breast cancer, colon cancer, pancreatic cancer, liver cancer, oral cancer, head and neck cancer and neuroblastoma[12-20]. It is thought that Periostin stimulates tumor cell growth by preventing apoptosis and promoting angiogenesis and enhances the survival of tumor cells via the Akt/PKB pathway[13,19]. Besides, Periostin always plays a great role in tumor invasion and metastasis[12,15,19].

In our recent study, we analyzed the samples of prostate biopsies from the patients with prostate cancer(PCa), benign prostate hyperplasia (BPH) and BPH with local prostatic intraepithelial neoplasm(PIN) by proteomics analysis using iTRAQ(Isobaric tags for relative and absolute quantification) combined with 2DLC-MS/MS (two-dimensional liquid chromatography-tandem mass spectrometry) to find the biomarkers of PCa. A total of 760 proteins were identified from 13787 distinct peptides. Among the 760 proteins, Prostate specific antigen and Prostatic acid phosphatase are well-known proteins enjoying clinical application. Based on the condition of screening differentially expressed proteins(the fold change cutoff ratio<0.66 or >1.50 as criterion to identify proteins of differential expression (P <0.05) was adopted), 20 proteins were significantly up-regulated and 26 were significantly down-regulated in the 116 labeled PCa samples compared with the 114 labeled BPH samples (Additional file 1, Table S1). Among the differentially expressed proteins, Periostin as the protein of interest was shown 9.12 fold up-regulation in PCa compared with BPH (Additional file 2, Figure S1)[21].

However, there are a little studies about the expression of Periostin in PCa. So, in our whole study, we focused on the expression and function of Periostin in PCa. The expression of Periostin was verificated by western blotting. The results revealed a significant increase of the amount of Periostin in PCa compared to BPH (Additional file 3, Figure S2B). Furthermore, immunohistochemical staining was performed to evaluate Periostin expression in the stromal or epithelial cells of prostate (Additional file 3, Figure S2A). Benign prostate glands expressed positive stromal Periostin in only 5/20 cases and positive epithelial Periostin in 8/20 cases; whereas the stroma of PCa was positive in 16/20 cases and the epithelium of PCa was positive in 12/20 cases. Statistical significance was observed for the stromal expression of Periostin between PCa and BPH (P <0.01). However, there was no statistical significance for the epithelial expression of Periostin between PCa and BPH (Additional file 4, Table S2)[21].

Here, Periostin was proposed to be a novel therapeutic target for PCa. Furthermore, the expression of Periostin in different PCa cell lines and the effect of sliencing Periostin by RNAi(RNA interference) on the proliferation and migration of PCa LNCap cells were studied.

## Materials and methods

### The identification and verification of Periostin

All the prostate biopsies from PCa, BPH and BPH with local PIN were analyzed by iTRAQ technology. Periostin was identified as a differential expressed protein of PCa compared to BPH and then the overexpression of Periostin in PCa was verificated by western blotting and immunohistochemical staining. The above processes have been reported by our group[21]. The details on the identification and verification of Periostin have been provided in the additional materials. The study was approved by the local ethics committee of Huashan Hospital of Fudan University.

### Cell culture

Human PCa cell lines:LNCap,DU-145,PC3,22RV1 were obtained from the Cell Bank of Chinese Academy of Sciences(Shanghai) and maintained in RPMI 1640 with 10% of fetal bovine serum, 100 u/mL of penicillin, and 50 mg/mL streptomycin (Beyotime, China) at 37°C in a 5% CO_2 _incubator. The cells were subcultured twice a week.

### shRNA lentiviral particles transfection

shRNA-Periostin lentiviral particles and control GFP lentiviral particles were obtained from Santa Cruz Biotechnology, Inc(USA). According to the instruction on the lentiviral particles, the cells were plated in a 12-well plate 24 hours prior to viral infection and incubated with 1 ml of complete optimal medium (with serum and antibiotics) overnight. The media in the plate wells was removed and replaced with 1 ml of Polybrene/media mixture(Santa Cruz,USA) per well. The cells were infected by adding the shRNA lentiviral particles to the culture. The plates were gently swirled to mix and incubate overnight. The stable clones expressing the shRNA cells were selected and divided 1:3 and subsequently incubated for 48 hours in complete medium. Then, the stable clones expressing the shRNA cells were selected via Puromycin dihydrochloride (Santa Cruz, USA).The culture medium was replaced with fresh puromycin-containing medium every 3-4 days until the resistant colonies can be identified. Several colonies were picked and analyzed for stable shRNA.

### Immunofluorescence staining for detecting efficancy of shRNA lentiviral particles transfection

Immunofluorescence staining was used for immunophenotype characterization of Periostin in different cell lines. The cells were fixed with 4% paraformaldehyde for 20 min, blocked with 5% bovine serum album for 45 min, then incubated with primary monoclonal antibody (1:200) at room temperature for 1 h. Cells were washed three times in PBS and incubated with corresponding secondary antibodies (1:200) for 2 h at room temperature. After second rinsing in PBS, The nuclei were stained with 4', 6-diamidino-2-phenylindole(DAPI, Sigma, USA) for 5 min at room temperature and then the cells were tested with fluorescence microscopy.

### Western blotting for detecting Periostin expression in PCa cell lines

The cells without treatment and the transfected cells were washed with PBS and harvested. Cell lysates were isolated by the protein extraction buffer (containing 150 mM NaCl, 10 mM Tris(pH 7.2), 5 mM EDTA, 0.1% Triton X-100, 5% glycerol, and 2% SDS), and then incubated at 4°C for 30 min. After centrifugation at 12,000 rpm for 30 min, the protein concentration in cell lysates was determined using Bradford assay. Proteins were denatured in sample buffer containing 2-mercaptoethanol and bromophenol blue for 10 min at 95°C. Equal amount of proteins (50 ug) was fractionated using 8 or 12% SDS-PAGE and transferred to PVDF membranes. After blocking with 5% non-fat milk, the membranes were incubated overnight at 4°C with the primary antibody. Then, the membranes washed with PBS three times were incubated in secondary antibody at room temperature. The intensity of target protein was detected using the enhanced chemiluminescence detection system.

### Reverse transcription PCR (RT-PCR) for detecting Periostin mRNA expression in PCa cell lines

Total RNA from PCa cell lines was extracted by the Trizol according to the instructions of manufacturer. The reverse transcription of RNA to cDNA was carried out using random primers of the SuperScript III First-Strand Synthesis SuperMix kit (Invitrogen).The forward and reverse primers were synthesized by Ying Jun Biotechn-ology,Inc (Shanghai) and presented as follows: Periostin (forward, 5' AGGCAAACAG CTCAGAGTCTTCGT 3' and reverse, 5' TGCAGCTTCAAGTAGGCTGAGGAA 3'). β-actin (forward, 5' CTGGCACCACACCTTCTACAATGA 3' and reverse, 5' TTAATGTCACGCACGATTTCCCGC 3'). For each pair of primers, the following protocol was applied. Initial denaturing: 2 minutes at 95°C, 40 cycles with denaturing at 94°C for 30 seconds, ananealing at Tm for 30 seconds and extension at 72°C for 1 minute. Products from PCR were separated by electrophoresis on a 2% agarose gel and then visualized witheth-idium bromide under ultraviolet light.

### Real-time PCR for detecting Periostin mRNA expression after shRNA lentiviral particles transfection

The procedures of the RNA extraction and the reverse transcription of RNA to cDNA are similar to the above description. Quant qRT-PCR (Sybr green I) Kit (Tiangen, Beijing) and qRT-PCR system (ABI, USA) were applied. The data was analyzed by ABI Prism 7300 SDS Software (ABI,USA) and the method of *ΔΔ*ct was used to calculate Periostin mRNA expression and the silence efficacy. The silence efficacy was determined by the formula: 1-2*^-ΔΔct^*.

### MTT assay

Cell proliferation was measured with the 3-(4,5-dimethylthiazol-2-yl) -2,5-diphenyl-ltetrazolium bromide (MTT, Sigma, USA) method. 200 μl of cells were seeded in a 96-well plate at a density of 4 × 10^3 ^cells per well and were subsequently incubated for 24 h to allow attachment. After incubation for 2,3,4,5,6 days, 20 μl MTT solution (5 mg/mL in PBS) were added to the wells for 4 h incubation before termination by aspiration of the media. The cells were then lysed with 150 μl dimethylsulfoxide (DMSO, Sigma, USA). The absorbance of the suspension was measured at 570 nm on an ELISA reader.

### Cell migration assay in vitro

The Millicell chambers (pore size 12 μm, insert size 12 mm, Millipore,USA) were set into 24-well plates which contained the supernatant of the cells(10^6^) for 48 h incubation. The Millicell chambers were removed from the well, and the matrigel was carefully removed from the membrane with a cotton wool stick. Then the Millicell was washed three times with PBS, fixed in 3% glutaral and stained with hematoxylin staining. The membrane was then removed from the Millicell, set upside down on a glass slide and covered with a coverslip. Cells were counted under the microscope at 200 × magnification. Eight fields were counted per membrane.

### Tumorigenicity in vivo

6-week-old male nude mice used for subcutaneous implantation of LNCaP cells were obtained from the Laboratory Animal Centre of Fudan University and housed in the laminar flow cabinets. Stably expressing shRNA-Periostin cells, control GFP cells and the cells without treatment were harvested and resuspended at 1 × 10^6^/100 μL in PBS. 500 μL suspension was then injected into the oxter of these mice (n = 6 for each group). Tumor growth was measured twice every week. After 42 days, all these mice were sacrificed and the tumors were dissected. The tissue slices from theses xenografts were analyzed by hematoxylin and eosin(HE) staining. The final tumor burden was measured by weight on the last day of the experiment. The size was determined by the formula: 0.5236L1(L2)^2^(L1:long diameter, L2:short diameter).

### Immunohistochemical staining for detecting Periostin expression in the xenografts

Immunohistochemical staining was performed to evaluate Periostin expression in these xenografts. Each slide was deparaffinized and rehydrated according to standard protocol, and treated with 10 mM sodium citrate buffer in a microwave pressure cooker at 120°C for 15 min. Sections were then immersed in 3% hydrogen peroxide and nonspecified binding was blocked in 5% normal goat serum. A polyclonal anti-Periostin was diluted 1:100. Immunohistochemical staining was conducted following the avidin-biotin peroxidase complex method with diaminobenzidine as a chromogen. Slides were counterstained with haematoxylin, dehydrated and mounted. Brown cytoplasmic staining of stromal or tumor cells was considered positive.

### Western blotting for detecting Periostin expression in the xenografts

To determine Periostin expression, the fresh tissue samples of these xenografts were analyzed by western blotting. The tissue samples were lysed in the protein extraction buffer (150 mM NaCl, 10 mM Tris(pH 7.2), 5 mM EDTA, 0.1% Triton X-100, 5% glycerol, and 2% SDS) after tripsis in liquid nitrogen and then incubated at 4 °C for 30 min. After centrifugation at 12,000 rpm for 30 min, the protein concentration in tissue homogenate was determined using Bradford assay. The processes of western blotting are similar to the above description.

### Statistics

The results are expressed as Mean±SD. Statistical analysis was performed using t-test or *X^2^*-test by SPSS 13.0. The difference is considered statistically significant when the P value is <0. 05.

## Results

### The expression of Periostin in PCa cell lines

**Figure 1:**
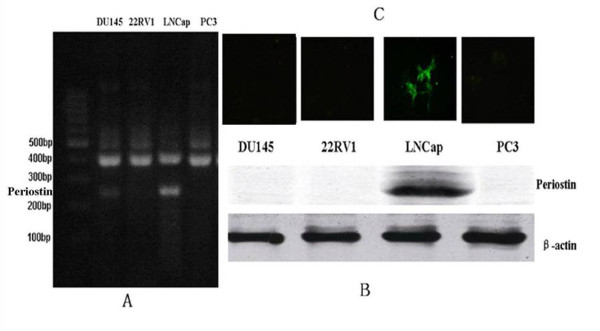
**The expression of Periostin in different PCa cell lines**. A: The results of RT-PCR analysis showed that the expression of Periostin mRNA was only detected in LNCap cells. B: The similar expression of Periostin protein in LNCap cells were confirmed by Western blot assay. C: The immunofluorescence staining indicated that green fluorescence only presented in LNCap cells and the other cell lines were negative.

The immunofluorescence staining showed that all the cell lines were negative except for the LNCap cells (Figure 1C). Similar results were confirmed by western blotting. Periostin was not detected in any of prostate cell lines, except for LNCap cells (Figure 1B). Concerning the expression of periostin mRNA in PCa cell lines, RT-PCR analysis showed a consistency with the expression of periostin protein (Figure 1A).

### The efficacy of shRNA lentiviral particles transfection

**Figure 2:**
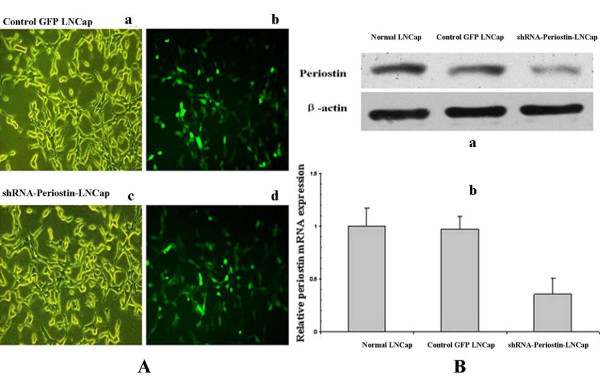
**The efficacy of shRNA lentiviral particles transfection**. A: Compared with LNCap cells transducted with the control GFP lentiviral particles (a) and shRNA-Periostin lentiviral particles(c) under common microscope, it could be seen that the effective rate of trusduction was more than 90% under fluorescence microscope(b and d). B: a:The Periostin protein expression was significantly reduced by shRNA-Periostin(P <0.05). b:The level of mRNA of Periostin after transducting shRNA-Periostin lentiviral particles was decreased by nearly 80% (P <0.05). Control GFP lentiviral particles had no influence on the Periostin expression of LNCap cells.

LNCap cells were chosen to continue the research of sliencing Periostin. The shRNA-Periostin lentiviral particles and control GFP lentiviral particles were directly obtained stably expressing among which cells with stable expression were identified (Figure 2A). LNCap cells transfected with the lentiviral particles showed green fluorescence under the fluorescence microscope. Both kinds of the infected LNCap cells showed above 90% transfection efficacy (Figure 2A). Real-time PCR was used to analyze the level of Periostin mRNA after transfecting shRNA lentiviral particles to LNCap cells. Figure 2B-b indicated that Periostin mRNA level of LNCap cells which stably expressed shRNA-Periostin was decreased by nearly 80% compared with the LNCap cells without treatment while control GFP lentiviral particles had no influence on the Periostin mRNA level of LNCap cells. As was expected, the Periostin protein expression was significantly reduced by shRNA-Periostin lentiviral particles (Figure 2B-a).

### Sliencing Periostin inhibits the proliferations of LNCap cells in vitro and in vivo

**Figure 3:**
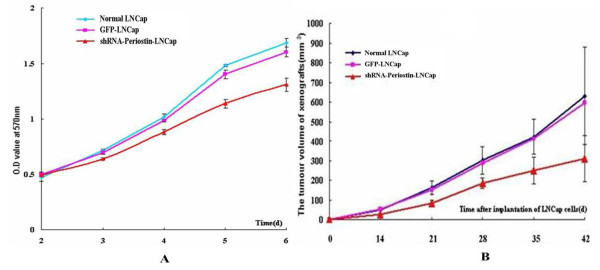
**Sliencing Periostin inhibits the proliferations of LNCap cells in vitro and vivo**. A: Growth curves of LNCap cells by MTT. Red represented LNCap cells transfected with shRNA-Periostin lentiviral particles. Green and pink represented normal LNCap cells and control GFP LNCap cells,respectively. From day 3, the stably expressing shRNA-Periostin LNCap cells started to grow slowly(P <0.05). B: The stably expressing shRNA-Periostin LNCap cells grew slowly in vivo. Red represented LNCap cells transfected with shRNA-Periostin lentiviral particles. Blue and pink represented normal LNCap cells and Control GFP LNCap cells, respectively.

To study the influence of sliencing Periostin on cell proliferation in vitro, we drew cell growth curves of LNCap cells based on the results of MTT. The results illustrated that the stably expressing shRNA-Periostin LNCap cells started to grow slowly from the third day (Figure 3A). There was significant difference in growth rates on 3 4,5,6 days compared with normal LNCap cells and control GFP LNCap cells (Figure 3A).

**Figure 4:**
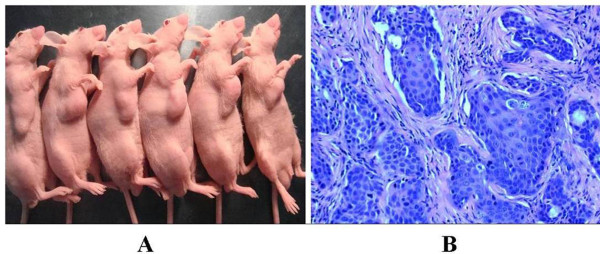
**The subcutaneous xenografts of LNCaP cells**. A: The nude mice with the subcutaneous xenografts of LNCaP cells were successfully established. The 6 nude mice listed belongs to the group of subcutaneous xenografts of normal LNCaP cells The subcutaneous xenografts can be seen in the oxter of the nude mice. B: The HE staining of these xenografts showed that the typical tumor cells of PCa scattered in clusters or nests with the enlarged and atypia nuclei containing prominent nucleoli which were isolated by redudant tumor-stroma.

Furthermore, to determine the effects of sliencing Periostin on LNCap cells in vivo, down-regulated Periostin LNCap cells, normal LNCap cells and control GFP LNCap cells were implanted into the oxter of the nude mice. After 42 days, the apparente tumors could be seen in the oxter of all these mice and no mouse was died(Figure 4A). After sacrificing these mice and dissectting the tumors, the tissue slices from theses xenografts were analyzed by HE staining. The HE staining of these xenografts showed that the typical tumor cells of PCa scattered in clusters or nests with the enlarged and atypia nuclei containing prominent nucleoli which were isolated by redudant tumor-stroma(Figure 4B)

**Figure 5:**
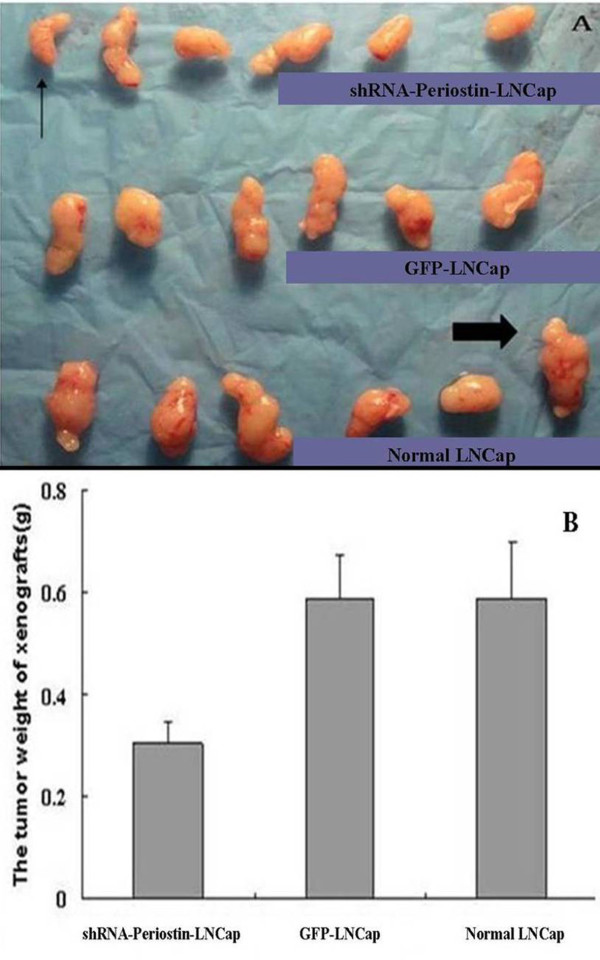
**The burden of the xenografts**. A:The tumors of the xenografts were listed, micr-arrow represented the minimum tumor and pykno-arrow represented the maximum tumor. B: The mean burden of tumors was minimum in the group of LNCap cells with down-regulated Periostin(P <0.05).

The growth curves of the tumors illustrated that the stably expressing shRNA-Periostin LNCap cells also grew slowly in vivo (Figure 3B). As shown in Figure 5A, the mean size of the tumors in the group of down-regulated Periostin LNCap cells was significantly smaller than the other two groups. The minimum tumor could been seen in the group of down-regulated Periostin LNCap cells and the maximum tumor could be found in the group of normal LNCap cells(Figure 5A). Sliencing Periostin of LNCap cells also resulted in a significant decrease in the tumor burden (Figure 5B).

### The expression of Periostin in the xenografts

**Figure 6:**
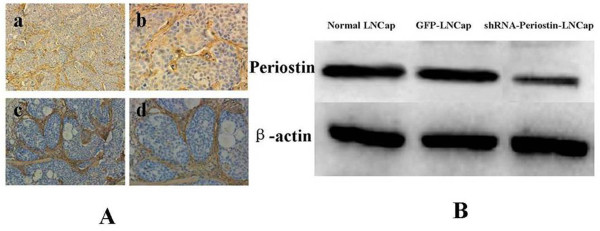
**The expression of Periostin in the xenografts**. **A: **The tissues of 12 xenografts from the groups of normal LNCap cells and control GFP-LNCap cells expressed strong positive Periostin in the stroma and tumor cells (a ×100, b ×200). But, the tissues of 6 xenografts from the group of down-regulated Periostin LNCap cells showed strong positive Periostin expression in the stroma and weak positive Periostin expression in tumor cells(c ×100, d ×200). B: The expression level of Periostin in the xenografts from the group of down-regulated Periostin LNCap cells significantly decreased compared to the xenografts from the other two groups.

Immunohistochemical staining was performed to evaluate Periostin expression in the stromal or tumor cells of the xenografts. The tissues of all 18 xenografts expressed strong positive Periostin in the stroma(Figure 6A) and the tissues of 12 xenografts from the groups of normal LNCap cells and control GFP-LNCap cells also expressed strong positive Periostin in the tumor cells(Figure 6A-a,6b). But, the tumor cells of the tissues of 6 xenografts from the group of down-regulated Periostin LNCap cells showed weak positive Periostin expression(Figure 6A-c,6d). Furthermore, the relative expression level of Periostin was detected by western blotting. The results revealed a significant decrease of the amount of Periostin in the xenografts from the group of down-regulated Periostin LNCap cells compared to the xenografts from the other two groups (Figure 6B).

### Sliencing Periostin inhibits migration of LNCap cells in vitro

**Figure 7:**
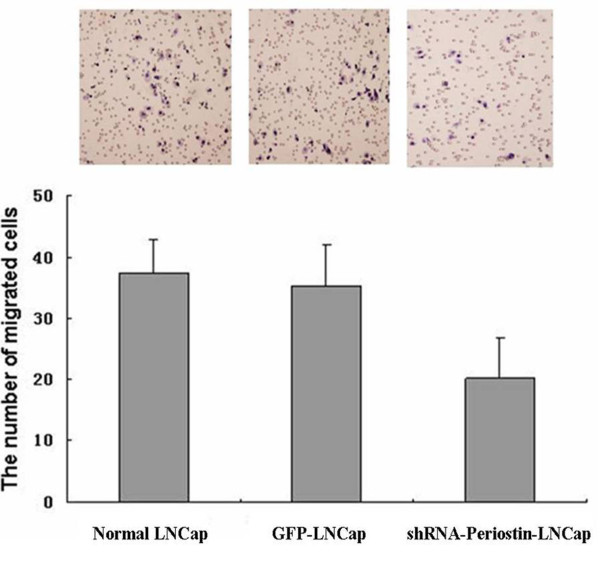
**The migration of LNCap cells was detected by Millicell**. The number was 37.38 ± 5.53, 35.38 ± 6.57, respectively for the nomal LNCap cells and control GFP LNCap cells. But, the number of migrated cells was 20.25 ± 6.71 in the group of LNCap cells with down-regulated Periostin(P <0.05).

To calculate the number of migrated cells stained with hematoxylin on the underside of the Millicell by microscope. For the LNCap cells of down-regulated Periostin, the number of migrated cells was 20.25 ± 6.71. For the normal LNCap cells and control GFP LNCap cells, the number was 37.38 ± 5.53 and 35.38 ± 6.57 respectively (Figure 7).The results indicated that sliencing Periostin significantly inhibited migration of LNCap cells in vitro (P <0.05).

## Discussion

The development of proteomics may help us better understand the pathological pathways of diseases and identify more promising targets. iTRAQ was developed by Applied Biosystems Incorporation in 2004. It labels global peptide, preserves post-translational modification information and makes quantitative proteomics analysis of 4 samples simultaneously under the same experimental conditions, compared with other approaches such as 2-DE (two-dimensional gel electrophoresis), ICAT (isotopecoded affinity tags) and SILAC (stable isotope labeling by amino acids in cell culture)[22,23]. The results of our recent study indicated a strong proof of the reliability of iTRAQ approach in the proteomics analysis of PCa. Periostin as an up-regulated protein has been found to be overexpressed in the stroma of PCa. Additionally, the correlation between Periostin and PCa has been studied. Tsunoda etal [24] defined gene expression signatures that are associated with 3-dimensional culture of prostate epithelial cells and extracted Periostin gene which was further evaluated using clinically PCa specimens. Their results demonstrated that Periostin expression was increased in the early stages of PCa (Gleason score 6-7), but not in the advanced stages of PCa. Furthermore, the positive ratio observed for the expression of PCa in tumor stroma was significantly correlated with the degree of malignancy. Tischlel etal [25] determined Periostin expression in the stromal and epithelial compartment of PCa, as well as the correlation with clinical data including patient follow up data in a larger cohort. Their results revealed that increased periostin expression in carcinoma cells was significantly associated with high Gleason score and advanced tumor stage. Additionly, the high stromal periostin expression was associated with higher Gleason scores and shortened PSA relapse free survival times. All the results of the above studies including ours indicate that periostin may be not only a promising biomarker for the prognosis of PCa but also a potential target for therapeutical intervention[21,24,25].

Periostin overexpression in human tumors can enhance tumor growth and always increase tumor invasion and metastasis[9,12]. The goal of our study is to observe the effect of sliencing Periostin by RNAi on the proliferation and migration of human PCa cell lines. RNAi is the sequence-specific gene-silencing induced by double-stranded RNA (dsRNA), and gives information about gene function in a quick, easy and inexpensive manner[26]. The shRNA(short-hairpin RNA) is widely used to induce RNAi in vertebrate cells, providing a tool to create continuous cell lines in which suppression of a target gene can be stably maintained[27]. Recently, many researchers have used plasmid and viral vectors for shRNA transcription, both in vitro and in vivo[26]. In our study, synthesized shRNA-Periostin lentiviral particles as a pool of concentrated, transfection-ready viral particles contain 3 target-specific constructs that encode 19-25nt (plus hairpin) shRNA designed to knock down gene expression at an efficacy of over 90%. Western blotting and Real-time PCR assays were used to evaluate the Periostin expression at protein level and mRNA level after transfection. Periostin mRNA level of LNCap cells with stably expressed shRNA-Periostin was decreased by nearly 80% compared with that of the LNCap cells without treatment. As was expected, the significantly lower level of the Periostin protein caused by shRNA-Periostin lentiviral particles was consistent with the change of Periostin mRNA level (Figure 2B).

Several studies have indicated that Periostin mRNA and protein are not expressed in several human cancer cell lines [11,28,29]. In our study, four different PCa cell lines: DU145, PC3, 22RV1 and LNCap were used to evaluate the expression of Periostin in PCa cells. Our results indicated that Periostin mRNA and protein were only expressed in the PCa LNCap cell line (Figure 1). LNCap cell line was isolated in 1977 by Horoszewicz et al from a needle aspiration biopsies of the left supraclavicular lymph node of a 50-year-old Caucasian male with confirmed diagnosis of metastatic prostate carcinoma. The LNCap cells responsive to 5-alpha-dihydrotestosterone can produce prostatic acid phosphatase and prostate specific antigen[30]. So, LNCap cell line is the best PCa cell line which can simulate biological behavior of PCa. The expressed differences of Periostin in PCa cell lines may be caused by different biological characteristics of those cell lines.

Though Periostin can promote the proliferation and the survival of several human cancer cell lines in vitro by inducing Akt/PKB pathway[12]. Some studies demonstrate that Periostin overexpression does not promote proliferation of human cancer cell lines including 293T, B16F1,MDA-MB-231,HSC2 and HSC3[4]. In our study, we have found that both the protein and mRNA of Periostin were only expressed in the PCa LNCap cell line(Figure 1). As a follow-up, we tried to explore the effect of sliencing Periostin on the proliferation of LNCap cells. MTT assay in vitro and tumorigenicity in vivo were used to evaluate the effect. As a result, stably expressing shRNA-periostin LNCap cells growed slowly in vitro and in vivo (Figure 3), which indicated that sliencing Periostin inhibited the proliferation of LNCap cells in vitro and in vivo.

The expression of Periostin in the xenografts was determined by immunohistocheical staining and western blotting. As a result, the weak positive Periostin expressed tumor cells could be seen in the tissues of 6 xenografts from the group of down-regulated Periostin LNCap cells which had a significant decrease of the amount of Periostin compared to the other two group(Figure 4). So, The decreased expression level of Periostin in the xenografts from the group of down-regulated Periostin LNCap also indicated the effect of RNAi in vivo. Additionally, the strong positive stromal Periostin expression in the tissues of all 18 xenografts revealed tumor-stroma interaction. Epithelial-mesenchymal transition (EMT), an important form of tumor-stroma interaction, plays a great role in tumor invasion and tumor metastasis [31]. Periostin has been reported to correlate with the process and facilitate the migration of the cancer cells[18]. According to our results, sliencing Periostin could inhibit migration of LNCap cells in vitro (Figure 5) which in turn may be involved in the change of EMT.

## Conclusion

Periostin as an up-regulated protein in PCa was identified by proteomics analysis of the samples of prostate biopsy, and then its overexpression in the stroma of PCa was confirmed in our recent study. Here, our study indicates that Periostin is only expressed in LNCap cell line and stably expressing shRNA-Periostin LNCap cells can be obtained by transfecting shRNA-Periostin lentiviral particles. Sliencing Periostin expression by RNAi can inhibit the proliferation and migration of LNCap cells. Therefore, Periostin may be a promising target of therapeutical intervention for PCa in future.

## A list of abbreviations used in the paper

2DLC-MS/MS: two-dimensional liquid chromatography-tandem mass spectrometry; BPH: benign prostate hyperplasia; HE: hematoxylin and eosin; iTRAQ: isobaric tags for relative and absolute quantification; PAP: prostatic acid phosphatase; PCa: prostate cancer; PIN: prostatic intraepithelial neoplasm; PSA: prostate specific antigen; RNAi: RNA interference; shRNA: short-hairpin RNA.

## Competing interests

The authors declare that they have no competing interests.

## Authors' contributions

CS and XZ carried out the studies and were co-first author. GX and QD participated in the design of the study. KX helped to draft the manuscript. JG and WL helped to finish the studies. WD and YG collected the samples. CS drafted the manuscript. All authors read and approved the final manuscript.

## Supplementary Material

Additional file 1 **Table S1. Differentially expressed proteins between 116(PCa) and 114(BPH)**. Based on the condition of screening differentially expressed proteins (the fold change cutoff ratio<0.66 or >1.50 as criterion to identify proteins of differential expression (P <0.05) was adopted), 20 proteins were significantly differentially up-regulated and 26 were significantly down-regulated in the 116 labeled PCa samples compared with the 114 labeled BPH samples. Click here for file

Additional file 2 **Figure S1. A representative MS/MS spectrum of Periostin**. The relative ratios of Periostin between 116(PCa) and 114(BPH) was 9.12. Periostin was identified with 13 peptides above the 95% confidence. This Figure displays the MS/MS spectrum of one peptide from Periostin. The peptide sequence: IITGPEIK is shown(The peptides above the 95% confidence are colored green and the peptides in the other colors have lower confidence). BPH samples were labeled with 114 tags, PCa samples were labeled with the 116 tags, and PIN samples were labeled with 117 tags. The peptide fragments including b-ion and y-ion series are shown in A and B. The quantitation information of the peptide is shown in C. Click here for file

Additional file 3 **Figure S2. The expression of periostin in malignant and benign prostate tissue**. A: Immunohistochemical staining of periostin in PCa and BPH. Negative epithelial and stromal periostin expression in BPH(a) and PCa(c). Positive epithelial and stromal periostin expression in BPH(b) and PCa(d). B: The results of western blotting revealed a significant increase of periostin amount in PCa compared to BPH(P <0.05). Click here for file

Additional file 4 **Table S2. Epithelial and stromal expression of periostin in PCa and BPH**. Benign prostate glands expressed positive stromal Periostin in only 5/20 cases and positive epithelial Periostin in 8/20 cases; whereas the stroma of PCa was positive in 16/20 cases and the epithelium of PCa was positive in 12/20 cases. Statistical significance was observed for the stromal expression of Periostin between PCa and BPH (P <0.01). However, there was no statistical significance for the epithelial expression of Periostin between PCa and BPH. Click here for file
