# Supplementary material for: Periostin: a promising target of therapeutical intervention for prostate cancer
Source: J Transl Med. 2011 Jun 30;9:99. doi: 10.1186/1479-5876-9-99 (PMC3146429; doi:10.1186/1479-5876-9-99)
Supplement: Additional file 1 — Table S1. Differentially expressed proteins between 116(PCa) and 114(BPH). Based on the condition of screening differentially expressed proteins (the fold change cutoff ratio<0.66 or >1.50 as criterion to identify proteins of differential expression (P <0.05) was adopted), 20 proteins were significantly differentially up-regulated and 26 were significantly down-regulated in the 116 labeled PCa samples compared with the 114 labeled BPH samples. [file 1479-5876-9-99-S1.DOC]

Supplementary table 1: Differentially expressed proteins between 116(PCa) and

114(BPH).

| Accession Protein name Peptides(95%) 116:114 | | | |
| --- | --- | --- | --- |
| Down-regulation | | | |
| sp|P17661 | Desmin | 62 | 0.03 |
| sp|O95810 | Serum deprivation-response protein 3 | 3 | 0.13 |
| sp|Q9BX66 | Sorbin and SH3 domain-containing protein 1 | 18 | 0.18 |
| sp|P08670 | Vimentin | 65 | 0.19 |
| sp|P51911 | Calponin-1 | 43 | 0.20 |
| sp|P04792 | Heat shock protein beta-1 | 15 | 0.22 |
| sp|P63267 | Actin, gamma-enteric smooth muscle | 191 | 0.24 |
| sp|Q969G5 | Protein kinase C delta-binding protein | 6 | 0.24 |
| sp|P12109 | Collagen alpha-1(VI) chain | 19 | 0.25 |
| sp|P11047 | Laminin subunit gamma-1 | 9 | 0.28 |
| sp|O94875 | Sorbin and SH3 domain-containing protein 2 | 2 | 0.32 |
| sp|P24821 | Tenascin | 7 | 0.35 |
| sp|Q15942 | Zyxin | 4 | 0.35 |
| sp|Q9Y490 | Talin-1 | 21 | 0.36 |
| sp|P02452 | Collagen alpha-1(I) chain | 92 | 0.38 |
| sp|P18206 | Vinculin | 15 | 0.39 |
| sp|P20774 | Mimecan | 6 | 0.40 |
| sp|Q93052 | Lipoma-preferred partner | 12 | 0.41 |
| sp|Q09666 | Neuroblast differentiation-associated protein  AHNAK | 62 | 0.42 |
| sp|P07585 | Decorin | 12 | 0.43 |
| sp|P98160 | Basement membrane-specific heparan sulfate proteoglycan core protein | 17 | 0.44 |
| sp|P30086 | Phosphatidylethanolamine-binding protein 1 | 7 | 0.47 |
| sp|P06396 | Gelsolin | 20 | 0.49 |
| sp|P12111 | Collagen alpha-3(VI) chain | 63 | 0.50 |
| sp|P01834 | Ig kappa chain C region | 4 | 0.53 |
| sp|P10909 | Clusterin | 3 | 0.54 |
| Up-regulation | | | |
| sp|P62241 | 40S ribosomal protein S8 | 2 | 1.53 |
| sp|P31943 | Heterogeneous nuclear ribonucleoprotein H | 3 | 2.17 |
| sp|P11021 | 78 kDa glucose-regulated protein | 27 | 2.21 |
| sp|P09669 | Cytochrome c oxidase polypeptide VIc | 2 | 2.38 |
| sp|P10606 | Cytochrome c oxidase subunit 5B,  mitochondrial | 7 | 2.70 |
| sp|P06576 | ATP synthase subunit beta, mitochondrial | 13 | 2.83 |
| sp|P38646 | Stress-70 protein, mitochondrial | 11 | 2.91 |
| sp|P21810 | Biglycan | 19 | 2.96 |
| sp|P49411 | Elongation factor Tu, mitochondrial | 6 | 3.02 |
| sp|P22314 | Ubiquitin-like modifier-activating enzyme 1 | 5 | 3.34 |
| sp|P13667 | Protein disulfide-isomerase A4 | 5 | 3.77 |
| sp|Q5SSJ5 | Heterochromatin protein 1-binding protein 3 | 5 | 4.06 |
| sp|P55327 | Tumor protein D52 | 3 | 4.25 |
| sp|Q99623 | Prohibitin-2 | 3 | 4.57 |
| sp|P07237 | Protein disulfide-isomerase | 17 | 4.70 |
| sp|Q9NZN4 | EH domain-containing protein 2 | 2 | 5.55 |
| sp|P10809 | 60 kDa heat shock protein, mitochondrial | 25 | 5.97 |
| sp|P06748 | Nucleophosmin | 10 | 7.94 |
| **sp|Q15063** | **Periostin** | **13** | **9.12** |
| sp|Q00796 | Sorbitol dehydrogenase | 9 | 9.82 |
