# Supplementary figures and images for: Periostin: a promising target of therapeutical intervention for prostate cancer
Source: J Transl Med. 2011 Jun 30;9:99. doi: 10.1186/1479-5876-9-99 (PMC3146429; doi:10.1186/1479-5876-9-99)

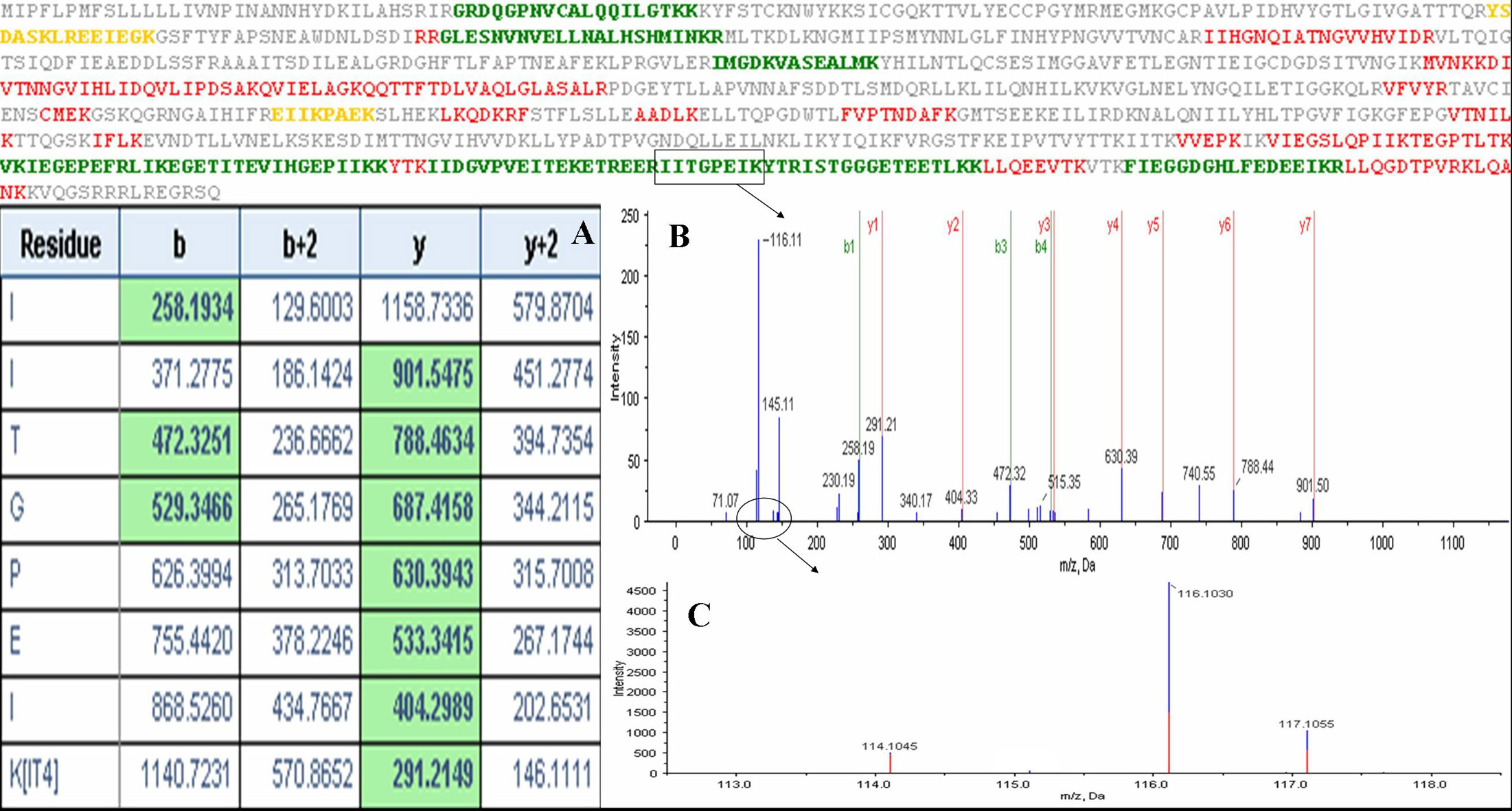

Supplement: Additional file 2 — Figure S1. A representative MS/MS spectrum of Periostin. The relative ratios of Periostin between 116(PCa) and 114(BPH) was 9.12. Periostin was identified with 13 peptides above the 95% confidence. This Figure displays the MS/MS spectrum of one peptide from Periostin. The peptide sequence: IITGPEIK is shown(The peptides above the 95% confidence are colored green and the peptides in the other colors have lower confidence). BPH samples were labeled with 114 tags, PCa samples were labeled with the 116 tags, and PIN samples were labeled with 117 tags. The peptide fragments including b-ion and y-ion series are shown in A and B. The quantitation information of the peptide is shown in C. [file 1479-5876-9-99-S2.JPEG]

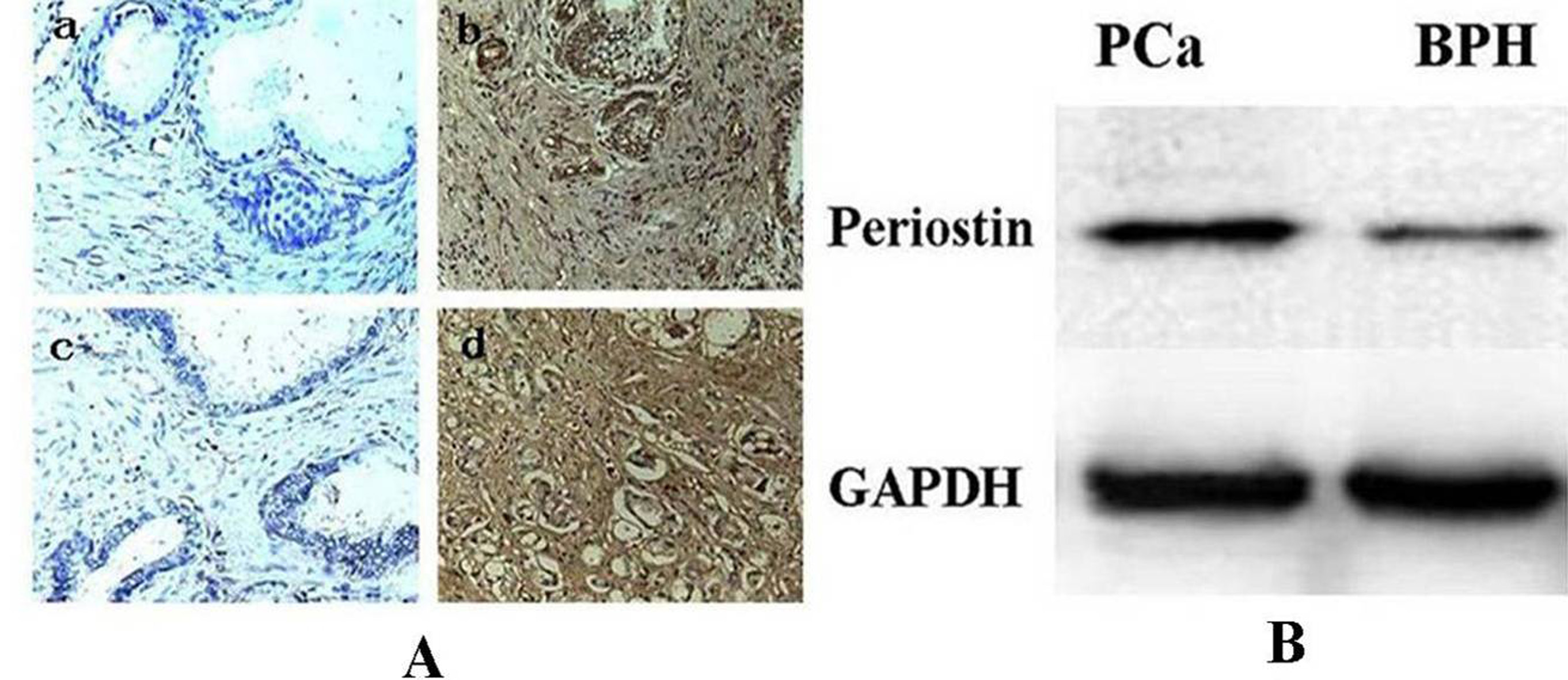

Supplement: Additional file 3 — Figure S2. The expression of periostin in malignant and benign prostate tissue. A: Immunohistochemical staining of periostin in PCa and BPH. Negative epithelial and stromal periostin expression in BPH(a) and PCa(c). Positive epithelial and stromal periostin expression in BPH(b) and PCa(d). B: The results of western blotting revealed a significant increase of periostin amount in PCa compared to BPH(P <0.05). [file 1479-5876-9-99-S3.JPEG]
