# Supplementary material for: Periostin: a promising target of therapeutical intervention for prostate cancer
Source: J Transl Med. 2011 Jun 30;9:99. doi: 10.1186/1479-5876-9-99 (PMC3146429; doi:10.1186/1479-5876-9-99)
Supplement: Additional file 4 — Table S2. Epithelial and stromal expression of periostin in PCa and BPH. Benign prostate glands expressed positive stromal Periostin in only 5/20 cases and positive epithelial Periostin in 8/20 cases; whereas the stroma of PCa was positive in 16/20 cases and the epithelium of PCa was positive in 12/20 cases. Statistical significance was observed for the stromal expression of Periostin between PCa and BPH (P <0.01). However, there was no statistical significance for the epithelial expression of Periostin between PCa and BPH. [file 1479-5876-9-99-S4.DOC]

Supplementary table 2: Epithelial and stromal expression of periostin in PCa and BPH

|  | Epithelial expression | |  | Stromal expression | |  |
| --- | --- | --- | --- | --- | --- | --- |
|  | Negative Positive P value | |  | Negative Positive P value | |  |
| PCa  BPH | 8 (40%)  12 (60%) | 12(60%)  8 (40%) 0.206 |  | 4 (20%) | 16(80%) |  |
|  |  |  |  | 15 (75%) 5 (25%) P<0.01 | |  |
